# Supplementary material for: Splice-Junction-Based Mapping of Alternative Isoforms in the Human Proteome
Source: Cell Rep. Author manuscript; Available in PMC 2020 Jan 15. (PMC6961840; doi:10.1016/j.celrep.2019.11.026)
Supplement: 3 [file NIHMS1546469-supplement-3.zip › DF2/PXD0010154/Spleen-209-Q9Y2Z2-VIQPGYGVQYDYLDPR.pdf]

A

Predicted sequence disorder and sequence features of Q9Y2Z2

Peptide: VIQPGYGVQYDYLDPR Junction: sp|Q9Y2Z2|MTO1_HUMAN|ENSG00000135297|SE2|40725|chr6|73480126|73480367|+1|r18|T1 TrNovel: FALSE

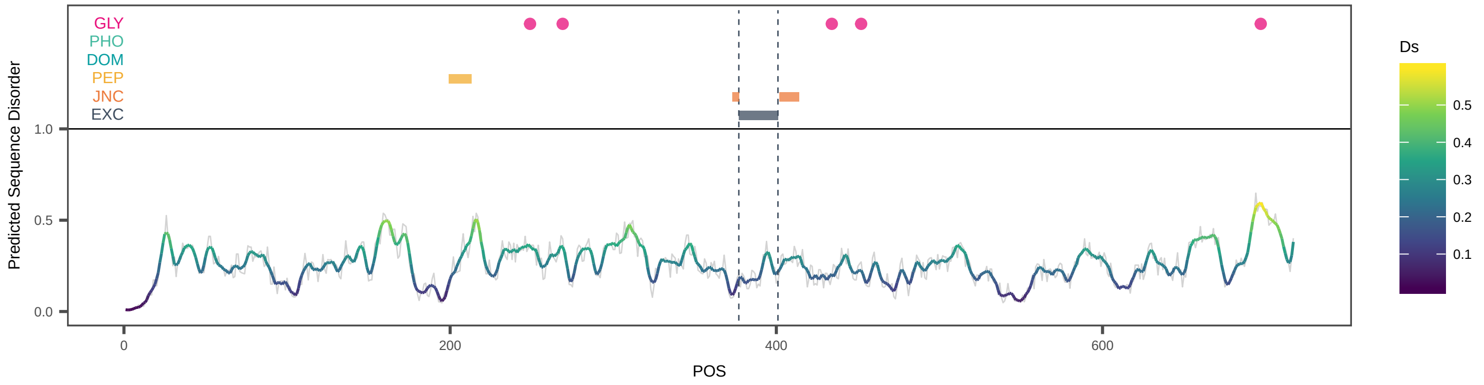

B

Distribution of sequence disorder in excised
vs. mapped and non−excised regions of protein

M−W P−value vs. mapped: 0.00916 vs. non−excised: 0.00344

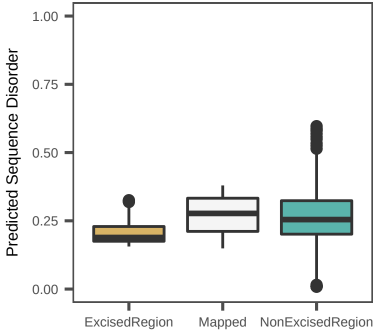

C
